# Balance Impairments in Adolescents Post‐Sports Concussion during Single and Dual Tasks

**DOI:** 10.1002/brb3.70502

**Published:** 2025-07-20

**Authors:** Abdulaziz A. Alkathiry, Anthony P. Kontos, Joseph M. Furman, Susan L. Whitney, Saud F. Alsubaie, Patrick J. Sparto

**Affiliations:** ^1^ Department of Physical Therapy and Health Rehabilitation, College of Applied Medical Sciences Majmaah University Majmaah Saudi Arabia; ^2^ Department of Orthopaedic Surgery University of Pittsburgh Pittsburgh Pennsylvania USA; ^3^ Department of Otolaryngology University of Pittsburgh Pittsburgh Pennsylvania USA; ^4^ Department of Physical Therapy University of Pittsburgh Pittsburgh Pennsylvania USA; ^5^ Department of Health and Rehabilitation Sciences, College of Applied Medical Sciences Prince Sattam bin Abdulaziz University Al‐Kharj Saudi Arabia

**Keywords:** concussion, postural stability, motor control

## Abstract

**Objective:**

This study aimed to explore changes in sway during single tasks and dual tasks in adolescents with and without sport concussion (SC).

**Methods:**

A cross‐sectional study of 57 adolescents with an SC and 67 healthy participants was compared on static balance during single‐ and dual‐task conditions on hard and compliant surfaces. Postural sway was assessed using a force platform (with and without a foam pad) during three cognitive conditions.

**Results:**

Individuals with SC had greater sway compared with healthy participants (*p* < 0.05). There were no significant interactions of groups and the task conditions. Across both groups, higher sway was observed in the compliant surface condition compared with the hard surface condition (*p* < 0.001). Greater sway was measured in the single task compared with the dual‐task conditions (*p* < 0.05), and the perceptual inhibition task generated greater sway than the spatial discrimination task (*p* < 0.05).

**Discussion:**

Several factors were associated with increased sway, including compliant surface, dual task, and perceptual inhibition tasks. However, the root‐mean‐square in the anteroposterior direction was the only measure that showed a difference between the two groups, while only the normalized path length sway measures illustrated the difference between the dual‐ and single‐task conditions.

## Graphical abstract

This study examined postural sway in 124 adolescents (57 with sports concussion, 67 healthy) during static balance tasks on hard and compliant surfaces under three conditions: no cognitive task, spatial discrimination, and perceptual inhibition. Adolescents with concussion exhibited significantly greater antero‐posterior root‐mean‐square (RMS) sway than healthy peers. Across all participants, sway increased on compliant surfaces and during perceptual inhibition tasks. Notably, normalized sway path length (NSPL) was reduced during dual‐task conditions compared to single‐task trials, possibly reflecting automatic stabilization or conscious compensation. These findings highlight that concussion‐related postural control deficits may be direction‐specific and task‐dependent. RMS sway in the antero‐posterior direction emerged as the most sensitive indicator of concussion‐related balance impairments. Clinically, incorporating dual‐task balance assessments and attention‐demanding tasks can enhance the detection of lingering deficits post‐concussion and support informed return‐to‐play decisions.

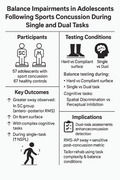

## 1 Introduction

Assessing concussion requires a multimodal assessment (Harmon et al. 2013; McCrory et al. 2013) of cognitive functioning and balance after concussion (Broglio et al. 2014; McCrory et al. 2013). Evaluating balance without considering tasks that require focused attention may mask underlying balance issues in concussed individuals, particularly in contexts where attention is diverted toward multiple objectives. This is particularly relevant in the context of contact sports, where athletes often face dynamic, complex scenarios that require the simultaneous management of multiple tasks, from maintaining stability to reacting swiftly to environmental stimuli. Neglecting to incorporate such attentional components in assessments may mask underlying balance deficits, especially in individuals with concussions who are returning to environments that demand high‐level multitasking (Dorman et al. 2015). Studies indicate that when postural and gait control tests are administered alongside another task, individuals with concussion exhibit sustained instability and imbalance compared to assessments conducted in isolation (Büttner et al. 2020; Catena et al. 2007; Dorman et al. 2015; Fait et al. 2013; Howell et al. 2019, 2013; Kleffelgaard et al. 2012). The effect of attention‐demanding tasks on concurrent balance performance in adolescents as measured using the center of pressure (CP) has not been extensively investigated in large samples at an early time point post‐concussion. Previous studies utilizing CP as an outcome measure have demonstrated its sensitivity in detecting postural instability following sports concussion (SC) (Corwin et al. 2020; Howell et al. 2020). Increased postural sway, particularly in the anteroposterior direction, has been observed in concussed individuals compared to healthy controls, with balance deficits often exacerbated under dual‐task conditions (Dorman et al. 2015; Rochefort et al. 2017). However, the interaction between concussion status, cognitive task complexity, and environmental stability in influencing postural control remains underexplored. Previous studies employing cognitive dual tasks have not examined the use of an inhibition test, a task known to be associated with balance performance (Redfern et al. 2009). Investigating how adolescents with SC adapt to different balance conditions under varying cognitive loads may provide further insight into the underlying mechanisms of post‐concussion postural instability.

The aim of this research is to investigate alterations in both single‐ and dual‐task balance abilities among adolescents with SC and healthy controls, utilizing laboratory‐based instruments such as force platforms. We postulated that adolescents affected by SC would demonstrate inferior balance performance, characterized by increased postural sway, in comparison to their healthy counterparts, particularly during dual‐tasking and under conditions of heightened balance challenge. These data are part of a larger research project from which a different question has been investigated (Eagle et al. 2021).

## 2 Methods

### 2.1 Study Design

This cross‐sectional study was designed to compare postural sway between two groups: individuals with SC and healthy participants. Postural sway was measured using the CP recorded from a force platform (BP5050, Bertec Inc., Columbus, OH) under two different conditions. In the first condition, participants stood directly on the force platform, providing a hard surface. In the second condition, they stood on a foam pad (Airex AG, Sins, Switzerland) placed on the force platform, creating a compliant surface. Each participant performed three trials on each surface: (1) standing without a cognitive task, (2) standing while performing a spatial discrimination visual task, and (3) standing while performing a perceptual inhibition visual task.

### 2.2 Participants

The study involved 124 adolescents aged 12 to 20 years, with a balanced distribution of males and females. This cohort included 57 participants (46%) with a symptomatic SC and 67 healthy controls (54%). Adolescents with SC were recruited from the UPMC Sports Medicine Concussion Clinic (a specialized concussion clinic) within 10 days post‐injury over an 18‐month period from 2015 to 2016. Healthy participants were sampled from the same community. Middle and high schools in the greater Pittsburgh area and from the University of Pittsburgh.

To be included in the SC group, participants had to meet specific diagnostic criteria: exhibiting symptoms (such as loss of consciousness, headache, amnesia, or dizziness) at the time of their concussion injury, showing declined neurocognitive test scores (Iverson et al. 2006), or experiencing worsened post‐concussion symptoms (Covassin et al. 2012). Concussion diagnoses were confirmed by licensed healthcare providers (e.g., neuropsychologists and physicians) based on medical history, clinical interviews, symptom reports, and assessments of cognitive, oculomotor, and vestibular impairments.

Exclusion criteria for all participants included a history of concussion within the past 6 months, neck pain or injury, lower body injuries affecting mobility, musculoskeletal disorders, brain surgery, substance abuse, major psychiatric or neurological disorders, vestibular disorders, special education needs, or a previous traumatic brain injury (TBI) with a Glasgow Coma Score < 13. Additionally, controls could not have a current symptomatic concussion or vestibular/balance disorder. The study received approval from the Institutional Review Board (IRB) at the University of Pittsburgh.

### 2.3 Procedures

Participants provided demographic information and medical and concussion history and completed the Post‐Concussion Symptom Scale (PCSS), which includes 22 self‐reported symptoms. The PCSS, consisting of 22 self‐reported symptoms, is a component of the Immediate Post‐Concussion Assessment and Cognitive Testing (ImPACT, ImPACT Applications, Pittsburgh, PA) computerized test (Iverson et al. 2003). They also rated current symptoms, such as headache, fogginess, dizziness, and nausea, on a scale from 0 to 10. Following these questionnaires, participants engaged in three experimental trials using an A–B–A block design (single task:dual task:single task). Each single‐task period lasted 20 s, while the dual‐task periods lasted 35 s for the spatial discrimination task and 75 s for the perceptual inhibition task. The cognitive tasks were performed in a fixed sequence (spatial discrimination first) for each surface condition, with the order of the surface conditions counterbalanced among participants to mitigate order effects.

The cognitive tasks were adapted from the Motor and Perceptual Inhibition Test (MAPIT) (Mohammad et al. 2010; Nassauer and Halperin 2003; Redfern et al. 2009) and were presented using E‐Prime 2.0 software (Psychology Software Tools, Pittsburgh, PA) on an LCD monitor positioned 125 cm from the participant. In the spatial discrimination task, participants pressed a thumb‐activated switch with either hand depending on the location of a rectangle on the screen. A total of 20 stimuli were randomly presented (average interval of 1.75 s), totaling 35 s. The perceptual inhibition task followed, requiring participants to press a switch based on the direction of an arrow on the screen. This task included 40 stimuli (20 congruous, 20 incongruous) presented at an average interval of 1.88 s, lasting 75 s in total. During congruous stimuli, the arrow's direction matched its position on the monitor, while in incongruous stimuli, the arrow appeared opposite to its actual direction (Alkathiry 2017). Before starting the sway assessment, participants practiced the cognitive tasks while seated to ensure understanding.

### 2.4 Data Collection and Analysis

The force platform measured vertical ground reaction forces and moments around the anteroposterior and mediolateral axes, with data sampled at 100 Hz using a custom LabVIEW program (National Instruments Corporation, Austin, Texas). These measurements were used to compute the CP.

The collected data were processed using a custom Matlab program (Mathworks, Inc. Natick, Massachusetts), where a low‐pass filter with a 2 Hz cutoff frequency (Sparto et al. 2004) was applied. This filter was chosen based on an examination of sway data frequencies, which indicated that a 2 Hz cutoff preserved 97% of the data.

Four measures of sway were calculated from the CP data: (1) root‐mean‐squared sway (RMS sway) in the anteroposterior direction (Equation 1). (2) RMS sway in the mediolateral direction (Equation 1). (3) Normalized sway path length (NSPL) in the anteroposterior direction (Equation 2). (4) NSPL in the mediolateral direction (Equation 2).

(1) $$\mathrm{RMS}-\mathrm{sway}\;={\left[\frac{\left[\sum_{n=1}^{N-1}\mathrm{Sway}{\left[n\right]}^{2}\right]}{N}\right]}^{\frac{1}{2}}$$

*N*: number of sway samples

Sway [*n*]: individual sway sample—the mean of all sway samples

(2) $$\mathrm{NSPL}\;=\left[\sum_{n=1}^{N-1}\left|\mathrm{Sway}\left[n+1\right]-\mathrm{Sway}\left[n\right]\right|\right]\;/\mathrm{duration}$$

N: number of sway samples

Sway [*n*]: individual sway sample

### 2.5 Statistical Analyses

To carry out the statistical analysis, SPSS software was employed, with the significance threshold set at *α* = 0.05. For comparing demographic variables between groups, the independent samples *t*‐test was used for data following a normal distribution, while the Mann–Whitney *U* test was applied for data not normally distributed. Dichotomous data were analyzed using chi‐square tests.

A linear mixed model with a compound symmetry covariance structure was adopted to examine the fixed effects of groups, balance conditions (single task and dual task), cognitive task types (spatial discrimination and perceptual inhibition), and surfaces (hard and compliant). Interaction effects between group and surface, group and balance condition, and group and cognitive task on the magnitude of four sway measures were also evaluated: the NSPL and the RMS‐sway in both the anteroposterior and mediolateral displacement of CP. The linear mixed model was selected due to its capability to handle study designs with missing data.

The sample size was determined using G*Power version 3.1.9 (Heinrich Heine University Düsseldorf, Düsseldorf, Germany) to ensure the detection of balance differences between groups. Using the partial *η* ^2^ value of 0.17 obtained from existing literature (Dorman et al. 2015), it was calculated that a minimum of 16 participants per group was required to detect statistically significant differences at *p* < 0.05.

## 3 Results

**FIGURE 1:**
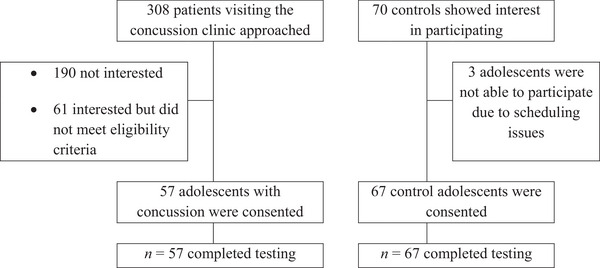
Subject enrollment flow chart.

Out of 308 adolescents visiting the concussion clinic, 57 (36 males, 63%) met the inclusion criteria and agreed to participate in the study, representing 18.5% of those approached. These participants had sustained an SC within the previous 10 days, with an average time since injury of 6.9 days (SD 2.4 days, range 0–10 days). Their ages ranged from 12 to 19 years, with a mean age of 15.0 years (SD 2.0 years). In addition, 67 healthy adolescents (37 males, 55%), with a mean age of 15.2 years (SD 2.3 years, range 12–20 years), were included. A flow chart (Figure 1) details the reasons for exclusion or nonparticipation.

The sports leading to injuries included soccer (*n* = 12), basketball (*n* = 10), football (*n* = 9), recreational activities (*n* = 6), hockey (*n* = 5), volleyball (*n* = 3), lacrosse (*n* = 3), wrestling (*n* = 2), softball (*n* = 2), and one case each for baseball, cheerleading, diving, rugby, and skiing. Adolescents with SC reported immediate symptoms post‐injury, with 46 out of 57 experiencing dizziness. Additionally, 30 out of 57 reported confusion or disorientation, 11 reported amnesia, and 10 reported LOC.

There were no significant differences between the concussion and healthy groups in terms of concussion history, migraine, attention deficit disorder, motion sickness, or learning disabilities. Symptom severity, assessed using the PCSS, revealed statistically significant group differences. Concussed participants had a mean symptom score of 27 (SD = 20), compared to 5 (SD = 9) for healthy participants. An independent samples *t*‐test indicated that this difference was statistically significant, *t*(122) = 8.10, *p* < 0.001. The mean difference between the groups was 22 (95% CI [16.62, 27.38]). Higher levels of headache, fogginess, dizziness, and nausea were reported by adolescents with concussions at recruitment compared to the healthy participants (Table 1).

**TABLE 1:** Characteristics and symptoms reported at the time of recruitment.

Characteristics/symptoms	Concussion group *n* = 57	Control group *n* = 67	*p* value
Age (years), mean (SD)	15.0 (2.0)	15.2 (2.3)	0.609^a^
Gender (male), *n* (%)	36 (63%)	37 (55%)	0.371^b^
Headache, mean (SD)	2.9 (1.6)	0.3 (0.6)	< 0.001^c^
Fogginess, mean (SD)	1.7 (1.7)	0.04 (0.2)	< 0.001^c^
Dizziness, mean (SD)	1.7 (1.6)	0.2 (0.5)	< 0.001^c^
Nausea, mean (SD)	0.9 (1.2)	0.1 (0.7)	< 0.001^c^

Abbreviation: SD, standard deviation.

^a^ independent samples *t* test.

^b^ Chi square test.

^c^ Kruskal–Wallis Test.

Incomplete data sets were noted for four adolescents in the concussion group and three in the healthy group in at least one sway measure. Significant main effects were observed for the group, balance condition, cognitive task, and surface in at least one of the four sway measures (Table 2).

**TABLE 2:** *p* values obtained from the linear mixed model for the effect of independent variables on center of pressure sway for adolescents with sports‐related concussion (*n* = 57) and controls (*n* = 67).

	NSPL		RMS‐sway	
*p* value of the effects	AP	ML	AP	ML
Group (concussion vs. control)	0.216	0.218	0.020^*^	0.136
Balance condition (single‐task vs. dual‐task)	< 0.001^*^	0.019 ^*^	0.587	0.282
Cognitive task (spatial discrimination vs. perceptual inhibition)	0.210	0.048^*^	< 0.001^*^	0.003^*^
Surface condition (hard vs. compliant)	< 0.001^*^	< 0.001^*^	< 0.001^*^	< 0.001^*^
Group by balance condition	0.591	0.658	0.125	0.264
Group by cognitive task	0.225	0.983	0.507	0.083
Group by surface	0.311	0.979	0.652	0.450

Abbreviations: AP, anterior‐posterior; ML, medial‐lateral; NSPL, normalized sway path length; RMS‐sway, root‐mean‐squared sway.

* *p* value < 0.05.

**FIGURE 2:**
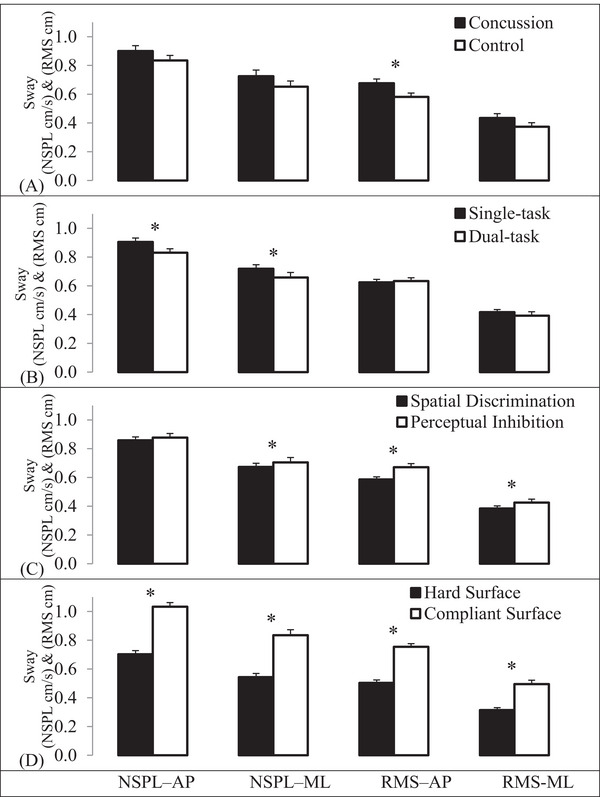
(A) Group, (B) Single versus dual‐task, (C) Cognitive task, and (D) Surface condition effects on the center of pressure. AP: anterior‐posterior; Error bars: standard deviation; ML: medial‐lateral; NSPL: normalized sway path length; RMS: root‐mean‐squared sway. *p < 0.05.

Significant main effects were observed for the group, balance condition, cognitive task, and surface in at least one of the four sway measures (Table 2). The main effect of the group indicated a significant increase in RMS sway in the anteroposterior direction (*p* = 0.02) for adolescents with concussions compared to healthy participants. However, no significant group effects were found for RMS sway in the mediolateral direction or NSPL in both directions (Figure 2A).

The balance condition effect showed a significant decrease in NSPL anteroposteriorly (*p* < 0.001) and mediolaterally (*p* = 0.019) during the dual tasks compared to the single task, though the RMS sway did not significantly differ (Figure 2B). The cognitive task effect revealed increased RMS sway in both directions during the perceptual inhibition task compared to the spatial discrimination task, along with a significant increase in NSPL in the mediolateral direction during perceptual inhibition (Figure 2C).

Increased sway was noted when participants stood on a compliant surface compared to a hard surface across all sway measures (Figure 2D). The results did not support a significant interaction between group and any of the task condition variables on CP.

## 4 Discussion

The objective of this study was to analyze postural sway changes in adolescents who had suffered an SC compared to healthy participants during single tasks and dual tasks. The key findings were: (1) adolescents with concussions exhibited a significant increase in anteroposterior RMS‐sway compared to healthy participants; (2) the dual task resulted in reduced NSPL compared to the single task; and (3) during the perceptual inhibition task, both RMS‐sway and NSPL increased compared to the spatial discrimination task. This study's strengths include a larger sample size than previous studies (Dorman et al. 2015; Rochefort et al. 2017) and the use of a novel perceptual inhibition attention task previously linked to balance performance (Redfern et al. 2009).

Our findings align with several earlier studies that reported differences in sway between concussed and healthy adolescents (Dorman et al. 2015; Guskiewicz 2001; Guskiewicz et al. 1996; Guskiewicz, Ross, et al. 2001). For instance, Guskiewicz et al. observed increased sway in adolescents with mild head injuries at 1 and 3 days post‐injury (Guskiewicz et al. 1996; Guskiewicz, Ross, et al. 2001), as well as 5 days post‐injury (Guskiewicz, Ross, et al. 2001). Similarly, a study reported sway differences between concussed adolescents and healthy participants within 10 days of injury (Dorman et al. 2015).

In this study, only RMS sway in the anteroposterior direction exhibited significant group differences, whereas RMS sway in the mediolateral direction or NSPL in both directions did not. This suggests that concussion‐related postural control deficits may be direction‐specific rather than uniformly affecting overall sway. Different sway measures vary in their sensitivity to balance impairments. RMS sway, in particular, has been identified as a sensitive indicator of postural control changes. Alkathiry (2025) highlighted its effectiveness in capturing dual‐task effects, with Martinez‐Mendez et al. (2012) demonstrating its ability to differentiate postural control between younger and older adults (Alkathiry 2025; Martinez‐Mendez et al. 2012). Furthermore, the variation between the measures aligns with research suggesting that the anteroposterior and the mediolateral sway respond differently to cognitive and postural demands (Tramontano et al. 2017).

These findings suggest that anteroposterior sway may be more sensitive to concussion‐related balance deficits in adolescents, whereas mediolateral sway and NSPL measures may be less reliable indicators in this context. Future research should further investigate which measures best capture clinically meaningful balance changes post‐concussion and how different cognitive or postural challenges influence these metrics.

Studies examining single‐ and dual‐task static balance in adolescents are comparable to our study (Dorman et al. 2015; Rochefort et al. 2017). Dorman et al. (2015) assessed balance performance in 18 adolescents with SC and 26 healthy participants at four time points post‐injury (approximately 10, 25, 49, and 78 days). At the 10‐day mark, adolescents with SC showed greater sway in both single‐ and dual‐task conditions, consistent with our results. However, at the second visit, increased sway was noted only during dual‐task conditions (Dorman et al. 2015). Rochefort et al. (2017) studied single‐ and dual‐task balance in 33 adolescents with SC and 33 healthy participants at approximately 30 days after concussion. Differences between the adolescents with SC and healthy participants appeared in both single‐ and dual‐task conditions for sway area but only for the dual‐task condition in sway velocity (Rochefort et al. 2017).

These findings suggest that CP‐based measures of sway differ between concussed and healthy adolescents within the first 10 days post‐injury during both single tasks and dual tasks. At later stages, dual tasks are more effective at distinguishing between groups. This is supported by recent meta‐analyses showing slower walking speeds during dual tasks in concussed individuals compared to healthy participants, even up to 2 months post‐injury (Büttner et al. 2020; Wood et al. 2019).

Regarding balance condition effects, a decrease in NSPL was observed during dual tasks compared to single tasks, which typically indicates enhanced balance control (Furman et al. 2013; Jacob et al. 2011; Pellecchia 2003; Ross et al. 2011; Teel et al. 2013).

This reduction aligns with studies involving healthy young adults who consistently showed lower postural sway during dual tasks (Jacob et al. 2011; Ross et al. 2011; Teel et al. 2013). However, a study by Pellecchia (2003) reported higher CP path length and variability in the anteroposterior direction during dual tasks. The discrepancy may be due to different sensory modalities affecting postural control. In our study, visual attention to an external focus during dual tasks may have stabilized postural sway compared to the auditory processing in Pellecchia's study, which was less integral to balance stabilization.

The finding of decreased NSPL but not RMS sway during dual tasks seems contradictory. RMS sway reflects deviation variability from the central point, while NSPL indicates higher frequency sway adjustments. During single tasks, participants might consciously try to control their balance, leading to increased NSPL due to higher frequency adjustments. Balance is usually maintained automatically without conscious effort, but deliberate control during assessments may interfere with automatic motor control, resulting in less efficient postural control (Vuillerme and Nafati 2007).

Engaging in a challenging perceptual inhibition task during balance resulted in increased sway in contrast to a simpler spatial discrimination task. This observation coincides with Pellecchia's (2003) findings, which demonstrated heightened sway during balancing tasks involving more demanding verbal activities. (Pellecchia 2003). This suggests that heightened attentional interference, especially with perceptual inhibition tasks, may reduce the attention available for balance regulation, leading to increased sway.

## 5 Limitations and Future Directions

A key limitation of this study is the differing durations of the cognitive tasks within the MAPIT test structure. Standardizing the duration of balance tests could improve the validity of comparisons across various cognitive tasks. Although clear instructions were given to maintain a standing position during balance tests, adolescents often became distracted during the single‐task conditions. To address this, continuous reminders, such as visibly displayed instructions, could help minimize distractions and maintain focus on the task. Another limitation is that our sample consisted solely of adolescents, which may not be representative of children or adult populations. Future research should aim to include a broader range of age groups to enhance the generalizability of the findings.

## 6 Conclusion

The key finding in this study was the observed differences in sway between adolescents with an SC within 10 days of injury and uninjured healthy participants. Factors associated with increased sway in both groups included the surface, whether the balance test was conducted alone or with a cognitive task, and the cognitive task complexity. These findings suggest that standard balance assessments may not fully capture postural instability, particularly in environments requiring cognitive engagement.

By integrating dual‐task balance assessments into clinical practice, healthcare providers can improve the sensitivity of concussion evaluations, facilitating early detection of postural control deficits that may otherwise go unnoticed. This approach can aid in individualized rehabilitation planning, ensuring that return‐to‐play decisions are informed by objective postural control measures rather than solely relying on symptom resolution.

Additionally, recognizing that specific task demands influence sway can help clinicians tailor rehabilitation strategies to address cognitive‐motor integration challenges in concussed adolescents.

## 7 Clinical Implications

These findings have important clinical implications for concussion assessment and rehabilitation. The RMS sway in the anteroposterior direction was more sensitive to concussion‐related balance deficits than the mediolateral direction or NSPL, suggesting that RMS sway in the anteroposterior direction should be prioritized in post‐concussion balance testing. Additionally, while both single‐ and dual‐task balance assessments detected group differences in the acute phase, dual‐task conditions may be more effective in identifying lingering impairments at later recovery stages. Clinicians should consider using dual‐task testing beyond the acute phase to track recovery progress and guide return‐to‐play decisions.

The increase in NSPL during single‐task conditions suggests that concussed adolescents may consciously overcompensate to maintain balance, leading to inefficient postural control strategies. Rehabilitation programs should incorporate dual‐task exercises that encourage automatic postural adjustments rather than excessive conscious corrections to facilitate a more natural recovery process.

Given that perceptual inhibition tasks resulted in greater sway than spatial discrimination tasks, it is clear that task complexity influences balance outcomes. This underscores the importance of developing more refined concussion balance testing protocols that integrate multiple sway measures, varying task complexities, and different postural conditions.

## Author Contributions

**Abdulaziz A. Alkathiry**: conceptualization, investigation, writing – original draft, methodology, writing – review and editing, formal analysis, data curation, visualization, software, funding acquisition, resources. **Anthony P. Kontos**: conceptualization, investigation, funding acquisition, validation, visualization, writing – review and editing, project administration, supervision, resources. **Joseph M. Furman**: conceptualization, validation, writing – review and editing, supervision. **Susan L. Whitney**: conceptualization, validation, writing – review and editing, supervision, resources. **Saud F. Alsubaie**: investigation, writing – review and editing, data curation. **Patrick J. Sparto**: conceptualization, investigation, methodology, validation, visualization, writing – review and editing, supervision, resources, software.

## Ethics Statement

University of Pittsburgh Institutional Review Board (IRB) approval was obtained for this study, with the assigned IRB number: PRO14060547.

## Conflicts of Interest

The authors declare no conflicts of interest.

## Peer Review

The peer review history for this article is available at https://publons.com/publon/10.1002/brb3.70502

## Data Availability

The data that support the findings of this study are available from the corresponding author upon reasonable request.
